# Understanding different trajectories of mental health across the general population during the COVID-19 pandemic

**DOI:** 10.1017/S0033291721000957

**Published:** 2021-03-03

**Authors:** Rob Saunders, Joshua E. J. Buckman, Peter Fonagy, Daisy Fancourt

**Affiliations:** 1 Centre for Outcomes Research and Effectiveness, Research Department of Clinical, Educational and Health Psychology, University College London, London, UK; 2 iCope – Camden and Islington Psychological Therapies Services, Camden & Islington NHS Foundation Trust, London, UK; 3 Research Department of Clinical, Educational and Health Psychology, University College London, London, UK; 4 Department of Behavioural Science and Health, University College London, London, UK

**Keywords:** Cohort study, COVID-19, lockdown, mental health outcomes, trajectories

## Abstract

**Background:**

The COVID-19 pandemic and nationally mandated restrictions to control the virus have been associated with increased mental health issues. However, the differential impact of the pandemic and lockdown on groups of individuals, and the personal characteristics associated with poorer outcomes are unknown.

**Method:**

Data from 21 938 adults in England who participated in a stratified cohort study were analysed. Trajectories of depression and anxiety symptoms were identified using growth mixture modelling. Multinomial and logistic regression models were constructed to identify sociodemographic and personality-related risk factors associated with trajectory class membership.

**Results:**

Four trajectories of depression and five for anxiety were identified. The most common group presented with low symptom severity throughout, other classes were identified that showed: severe levels of symptoms which increased; moderate symptoms throughout; worsening mental health during lockdown but improvements after lockdown ended; and for anxiety only, severe initial anxiety that decreased quickly during lockdown. Age, gender, ethnicity, income, previous diagnoses, living situation, personality factors and sociability were associated with different trajectories.

**Conclusions:**

Nearly 30% of participants experienced trajectories with symptoms in the clinical range during lockdown, and did not follow the average curve or majority group, highlighting the importance of differential trajectories. Young, female, outgoing and sociable people and essential workers experienced severe anxiety around the announcement of lockdown which rapidly decreased. Younger individuals with lower incomes and previous mental health diagnoses experienced higher and increasing levels of symptoms. Recognising the likely symptom trajectories for such groups may allow for targeted care or interventions.

## Introduction

The COVID-19 pandemic has had a profound impact on the emotional state of many people across the world, leading to fears of increased mental health burden (Gunnell et al., 2020; Holmes et al., 2020). Both the illness itself and governmental attempts to control the pandemic have exposed populations to the greater likelihood of experiencing stressful life events (Brooks et al., 2020; Luykx, Vinkers, & Tijdink, 2020) such as severe illness, bereavement, unemployment and debt (Fancourt, Steptoe, & Wright, 2020b; Greenberg, Docherty, Gnanapragasam, & Wessely, 2020; Hall et al., 2020; Takian, Raoofi, & Kazempour-Ardebili, 2020; Woolhandler & Himmelstein, 2020). All of these are associated with a greater likelihood of experiencing depression and other common mental health problems (Paykel, 2003), and a greater likelihood of recurrences of depression among those that have had prior depressive episodes (Monroe, Anderson, & Harkness, 2019). In addition, the large-scale ‘lockdowns’, ‘stay-at-home’ orders and quarantining requirements have led to increases in loneliness and social isolation (Bu, Steptoe, & Fancourt, 2020), which are also associated with greater risk of mental health problems (Wang, Mann, Lloyd-Evans, Ma, & Johnson, 2018).

General population studies have found that both psychological distress and the proportion of adults with clinically significant mental illness increased in the early weeks of the pandemic (Every-Palmer et al., 2020; Layard et al., 2020; Shevlin et al., 2020). Distress was higher among those with low incomes or that had lost income during lockdown, and those with pre-existing health conditions (Shevlin et al., 2020). However, there was a gradual decrease in symptoms of depression and anxiety in the UK general population during lockdown (Fancourt, Steptoe, & Bu, 2020a). Most studies so far have focused on the average symptom changes or trajectories, but this can obscure different patterns of experiences. There is emerging evidence that particular groups may have had different symptom trajectories across the pandemic. For example, a study of self-reported depression and anxiety symptoms in primary care mental health services found a brief increase in anxiety scores in the first 2 weeks of lockdown, followed by a return to normal levels and then a slight increase following the easing of lockdown restrictions (Saunders, Buckman, Leibowitz, Cape, & Pilling, 2021). Similarly, a qualitative study reported that individuals with pre-existing mental health conditions experienced worsening mental health during lockdown (Burton, McKinlay, Aughterson, & Fancourt, 2020).

It would appear that lockdown may have helped to stabilise anxiety and depressive symptoms for many, but it may have exacerbated symptoms of mental ill health for others. Similarly, even though many people adapted to the experience of lockdown and may even have experienced further improvements in mental health as lockdown lifted, the easing of lockdown may have posed new challenges for others, including disrupting newly-acquired routines and coping patterns. Therefore, this study aimed to identify differential trajectories of anxiety and depression symptoms before, during and after the easing of lockdown in England using growth mixture modelling (GMM), and explore participant characteristics associated with these trajectories, in order to determine how individuals have been affected and identify groups that may need additional support for their mental health.

## Methods

### Participants

Data were drawn from the COVID-19 Social Study, described in detail elsewhere (Bu et al., 2020; Fancourt et al., 2020a) and with further information provided in online Supplementary Appendix A. In brief, this was a large stratified panel study of the weekly psychological and social experiences of over 70 000 adults (aged 18+) in the UK during the COVID-19 pandemic. The study was approved by the UCL Research Ethics Committee (12467/005) and all participants gave informed consent.

We included participants recruited between the 21st March 2020 and 10th July 2020 residing in England that provided data at least three times before the end of the first UK national lockdown on 10th May (which started on 23rd March) and at least three times in the 8 weeks after the lockdown restrictions were eased. A small number of participants were missing data on gender, age, ethnicity, local level deprivation, and level of educational attainment, and therefore could not be weighted in analyses (see below). See online Supplementary eFigure. 1 for details of study flow.

To account for the non-random nature of the COVID-19 Social Study, the sample was weighted by the proportions of gender, age, ethnicity, education, and country of living obtained from the Office for National Statistics (Office for National Statistics, 2018).

### Measures

Participants were asked to complete measures assessing symptoms of depression (PHQ-9) (Kroenke, Spitzer, & Williams, 2001) and anxiety (GAD-7) (Spitzer, Kroenke, Williams, & Löwe, 2006) each week, and in addition at baseline the following were also recorded: age, gender, ethnicity, income, living situation, history of mental health and chronic physical health diagnoses, and personality (Big Five Inventory, BFI) (Soto & John, 2017). For further details on measures and participant characteristics see online Supplementary Appendix B and eTable 1.

### Analysis

In order to identify different trajectories of mental health symptoms before and after the easing of lockdown restrictions, piecewise GMM was employed. GMM accounts for heterogeneity in patterns of change by identifying statistically distinct trajectories, and therefore sub-groups of individuals displaying similar patterns of change (Muthén et al., 2002; Rubel et al., 2015). Piecewise models are used to model potentially non-linear effects as they specify the break point for separate linear components (Kohli & Harring, 2013). For the current analysis, the easing of lockdown restrictions was used as the break point for the trajectories.

Model fit of the GMM models were compared using the Vuong–Lo–Mendell–Rubin likelihood ratio test (Lo, Mendell, & Rubin, 2001), the Akaike information criterion, Bayesian information criterion, and entropy values, with full information about model selection decisions presented in online Supplementary Appendix C. Missing data were handled using full information maximum-likelihood through the expectation maximisation algorithm (Dempster, Laird, & Rubin, 1977), and survey weights were trimmed at the top 90% to minimise the impact of extreme weights (Asparouhov & Muthen, 2007). GMM analysis was conducted in Mplus version 8 (Muthén & Muthén, 2015).

Multinomial logistic regression models were then fitted to test associations between the different trajectory classes and baseline participant characteristics. This included factors found to be associated with increased risk of mental health distress during the COVID-19 pandemic: age, gender, employment, ethnicity, previous mental health diagnosis, being a keyworker, caring for others, previous amount of social contact, and personality characteristics.

## Results

### Descriptive statistics

A sample of 21 938 participants met the inclusion criteria. There was an over-representation of women (76%) and people with university degrees (70%), as well as an under-representation of people from Black, Asian and minority ethnic (BAME) groups (5%) compared to the general population (Table 1). Applying weighting resulted in a better reflection of the general population. The weighted sample was made up of 12 690 (58%) women, 1903 (9%) participants from BAME groups and 8913 (41%) with university degrees. There were 3768 participants (17%) reporting mental health diagnoses on entry to the study.

**Table 1.** Descriptive statistics

	Raw data		Weighted data	
	*N*	%	*N*	%
Gender				
Women	16 612	76	12 690	58
Men	5326	24	9248	42
Age in years				
18–29	1282	6	2681	12
30–45	5315	24	4203	19
46–59	7269	33	7523	34
60+	8072	37	7531	34
Ethnicity				
White	21 018	96	20 035	91
Black, Asian and minority ethnic (BAME) groups	920	4	1903	9
Household income (per annum)				
<£30 000	7557	34	8959	41
⩾£30 000	12 309	56	10 755	49
Prefer not to say	2072	9	2224	10
Keyworker				
No	17 445	80	17 532	80
Yes	4493	20	4406	20
Education				
General Certificate of Secondary Education (GCSE) or below	2898	13	6241	28
A-levels or equivalent	3760	17	6784	31
Undergraduate degree or above	15 280	70	8913	41
Carer				
No	18 547	85	18 715	85
Yes	3391	15	3223	15
Living status				
Alone	4519	21	4301	20
With others, without children	12 479	57	13	60
With others, including children	4940	23	4567	21
Overcrowded				
No	20 124	92	19 569	89
Yes	1814	8	2369	11
Urban/rural				
Rural	10 412	47	10 384	47
Urban	11 526	53	11 554	53
Diagnosed mental illness				
No	18 349	84	18 170	83
Yes	3589	16	3768	17
Long-term physical health condition				
No	13 012	59	12 632	58
Yes	8926	41	9306	42
Previous social contact frequency				
Every day	2342	11	2262	10
Three or more times a week	5701	26	5141	23
Once or twice a week	7580	35	7552	34
Once or twice a month	4066	19	4220	19
Less than once a month	2249	10	2764	13
Big Five personality factor	*M*	s.d.	*M*	s.d.
Neuroticism	11.14	4.24	11.20	4.35
Extraversion	12.83	4.26	12.62	4.26
Openness	15.39	3.25	14.90	3.27
Agreeableness	15.55	3.03	15.44	3.09
Conscientiousness	15.99	2.91	15.83	2.97

### Trajectories of depression and anxiety symptom change

**Fig. 1.**
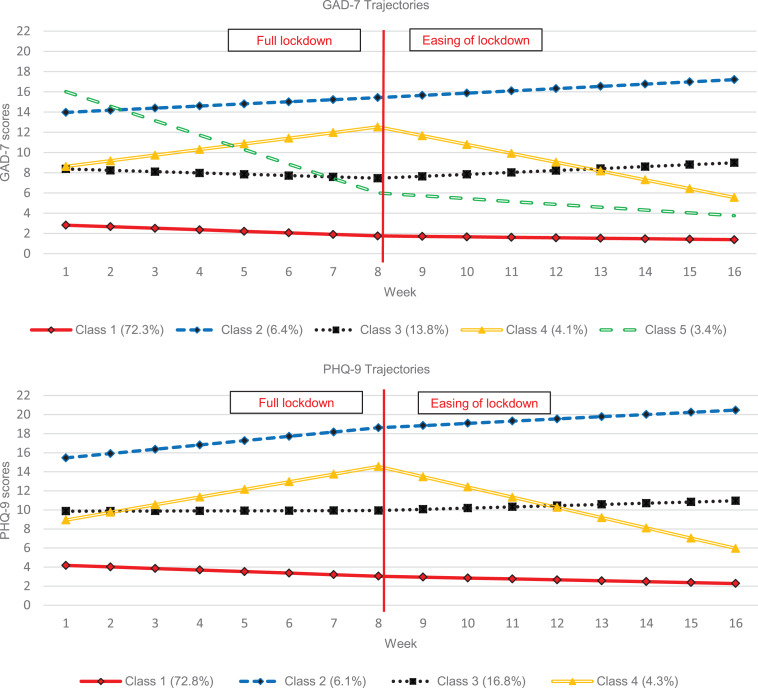
GMM class solution trajectories (GAD-7 and PHQ-9).

Piecewise GMM was performed on both the GAD-7 and PHQ-9 data independently to identify distinct trajectories of symptom score change. A total of 292 608 GAD-7 (mean = 4.11, s.d. = 4.81) and 292 860 PHQ-9 scores (mean = 5.4, s.d. = 5.43) were analysed, PHQ-9 and GAD-7 scores were highly correlated (*r* = 0.82, *p* < 0.001). Model fit statistics are presented in online Supplementary eTable 2, with the trajectories presented in Fig. 1. Classes 1–4 appear very similar between the two panels, but the fifth GAD-7 class appears distinct from the others. The classes are described as follows: class 1 (low symptom severity throughout with slight further improvements over time; the most common class), class 2 (moderate/moderately-severe symptoms that become severe over time), class 3 (moderate symptom severity, remaining relatively constant), class 4 (worsening mental health during lockdown but improvements after lockdown easing) and – for GAD-7 only – class 5 (severe initial anxiety that decreases to normal range, predominantly during lockdown).

### Associations with trajectory classes

Descriptive statistics of each identified anxiety and depression class are presented in online Supplementary eTable 3, and characteristics independently associated with the likelihood of membership to anxiety classes are presented in Table 2, and for depression classes in Table 3. Class 1 (low symptoms) is used as the reference group in these multinomial regression models.

**Table 2:** Associations between participant characteristics and GAD-7 trajectory classes

	Class 2 (vs Class 1)			Class 3 (vs Class 1)			Class 4 (vs Class 1)			Class 5 (vs Class 1)		
	RRR	*95% CIs*	*p*-value	RRR	*95% CIs*	*p*-value	RRR	*95% CIs*	*p*-value	RRR	*95% CIs*	*p*-value
Gender: Women (vs men)	1.12	(*0.97;1.29*)	0.137	**1**.**16**	(***1.06;1.28*)**	**0**.**002**	**1**.**56**	(***1.31;1.85*)**	**<0**.**001**	**2**.**04**	(***1.66;2.5*)**	**<0**.**001**
Age: 18–29 years (vs 60+ years)	**2**.**56**	(***2.01;3.25*)**	**<0**.**001**	**2**.**13**	(***1.81;2.5*)**	**<0**.**001**	**4**.**21**	(***3.2;5.53*)**	**<0**.**001**	**2**.**71**	(***2.03;3.62*)**	**<0**.**001**
Age: 30 to 45 years (vs 60+ years)	**2**.**03**	(***1.64;2.52*)**	**<0**.**001**	**1**.**70**	(***1.47;1.97*)**	**<0**.**001**	**3**.**09**	(***2.39;3.99*)**	**<0**.**001**	**1**.**90**	(***1.44;2.51*)**	**<0**.**001**
Age: 46 to 59 years (vs 60+ years)	**1**.**55**	(***1.29;1.87*)**	**<0**.**001**	**1**.**40**	(***1.24;1.58*)**	**<0**.**001**	**1**.**91**	(***1.52;2.4*)**	**<0**.**001**	**1**.**61**	(***1.27;2.05*)**	**<0**.**001**
Ethnicity: Black, Asian, Minority (vs White)	1.22	(*0.98;1.53*)	0.078	**1**.**32**	(***1.14;1.52*)**	**<0**.**001**	1.25	(*0.99;1.59*)	0.059	0.73	(*0.53;1.01*)	0.058
Education: Low (vs High)	**1**.**23**	(***1.02;1.47*)**	**0**.**026**	0.93	(*0.82;1.05*)	0.239	**1**.**23**	(***1;1.5*)**	**0**.**046**	1.21	(*0.96;1.51*)	0.104
Education: Medium (vs High)	1.15	(*0.98;1.35*)	0.096	0.90	(*0.81;1*)	0.056	0.85	(*0.71;1.02*)	0.080	1.20	(*0.99;1.46*)	0.065
Income: <£30,000 (vs >£30,000)	**1**.**63**	(***1.4;1.9*)**	**<0**.**001**	**1**.**20**	(***1.09;1.33*)**	**<0**.**001**	**1**.**29**	(***1.09;1.53*)**	**0**.**003**	1.14	(*0.95;1.37*)	0.160
Income: prefer not to say (vs >£30,000)	1.19	(*0.95;1.48*)	0.127	1.04	(*0.9;1.21*)	0.603	0.79	(*0.6;1.04*)	0.095	0.83	(*0.63;1.1*)	0.196
Alone (vs With others, no children)	1.13	(*0.95;1.34*)	0.162	**1**.**20**	(***1.07;1.34*)**	**0**.**002**	**1**.**24**	(***1.01;1.51*)**	**0**.**037**	0.91	(*0.72;1.15*)	0.433
Living with others, with children (vs Others, no children)	1.17	(*0.99;1.39*)	0.061	1.10	(*0.99;1.24*)	0.085	**1**.**27**	(***1.06;1.52*)**	**0**.**009**	1.14	(*0.94;1.39*)	0.191
Mental health diagnosis (vs none)	**5**.**73**	(***4.99;6.58*)**	**<0**.**001**	**2**.**83**	(***2.55;3.14*)**	**<0**.**001**	**3**.**15**	(***2.67;3.7*)**	**<0**.**001**	**2**.**08**	(***1.73;2.5*)**	**<0**.**001**
Carer (vs not a carer)	**1**.**26**	(***1.06;1.5*)**	**0**.**009**	**1**.**22**	(***1.08;1.37*)**	**0**.**001**	1.00	(*0.82;1.23*)	0.975	0.98	(*0.79;1.22*)	0.866
Keyworker (vs not a keyworker)	1.06	(*0.9;1.25*)	0.485	1.05	(*0.94;1.17*)	0.388	**1**.**33**	(***1.13;1.57*)**	**0**.**001**	**1**.**50**	(***1.25;1.8*)**	**<0**.**001**
Long-term health condition (vs none)	**1**.**84**	(***1.61;2.1*)**	**<0**.**001**	**1**.**33**	(***1.21;1.46*)**	**<0**.**001**	**1**.**30**	(***1.12;1.51*)**	**0**.**001**	**1**.**40**	(***1.18;1.65*)**	**<0**.**001**
Overcrowded living (vs not)	**1**.**31**	(***1.09;1.57*)**	**0**.**005**	**1**.**32**	(***1.16;1.5*)**	**<0**.**001**	1.04	(*0.84;1.29*)	0.717	1.03	(*0.81;1.31*)	0.811
Urban (vs Rural)	1.12	(*0.98;1.28*)	0.088	1.02	(*0.94;1.12*)	0.612	0.99	(*0.85;1.14*)	0.846	1.16	(*0.99;1.37*)	0.066
Social: every day (vs once/twice a week)	1.10	(*0.86;1.41*)	0.429	1.03	(*0.89;1.21*)	0.676	1.21	(*0.95;1.54*)	0.126	**1**.**73**	(***1.34;2.24*)**	**<0**.**001**
Social: three/four times a week (vs once/twice a week)	1.08	(*0.9;1.29*)	0.400	0.90	(*0.8;1.01*)	0.071	0.86	(*0.7;1.04*)	0.117	1.19	(*0.96;1.47*)	0.120
Social: once/twice a month (vs once/twice a week)	1.15	(*0.96;1.38*)	0.117	1.02	(*0.91;1.15*)	0.738	**0**.**80**	(***0.65;0.99*)**	**0**.**039**	**1**.**28**	(***1.03;1.6*)**	**0**.**027**
Social: less once month (vs once/twice a week)	**1**.**62**	(***1.34;1.96*)**	**<0**.**001**	**1**.**27**	(***1.11;1.46*)**	**0**.**001**	1.25	(*1;1.58*)	0.053	1.21	(*0.92;1.59*)	0.178
Personality: Neuroticism	**1**.**42**	(***1.39;1.45*)**	**<0**.**001**	**1**.**23**	(***1.21;1.24*)**	**<0**.**001**	**1**.**26**	(***1.23;1.28*)**	**<0**.**001**	**1**.**37**	(***1.34;1.4*)**	**<0**.**001**
Personality: Extraversion	1.00	(*0.98;1.02*)	0.930	**1**.**02**	(***1;1.03*)**	**0**.**007**	**1**.**05**	(***1.03;1.07*)**	**<0**.**001**	**1**.**04**	(***1.02;1.06*)**	**<0**.**001**
Personality: Openness	**1**.**07**	(***1.05;1.09*)**	**<0**.**001**	**1**.**04**	(***1.03;1.06*)**	**<0**.**001**	**1**.**05**	(***1.03;1.07*)**	**<0**.**001**	**1**.**08**	(***1.05;1.1*)**	**<0**.**001**
Personality: Agreeableness	**0**.**97**	(***0.95;0.99*)**	**0**.**001**	1.01	(*0.99;1.02*)	0.207	0.99	(*0.96;1.01*)	0.271	1.00	(*0.97;1.02*)	0.745
Personality: Conscientiousness	**1**.**05**	(***1.02;1.07*)**	**<0**.**001**	**0**.**98**	(***0.97;1*)**	**0**.**011**	1.02	(*1;1.05*)	0.083	**1**.**05**	(***1.02;1.08*)**	**0**.**002**

Notes: RRR = relative risk ratio; 95%CIs = 95% confidence intervals. Figures in bold are statistically significant at *p* < 0.05.

**Table 3.** Associations between participant characteristics and PHQ-9 trajectory classes

	Class 2 (vs Class 1)			Class 3 (vs Class 1)			Class 4 (vs Class 1)		
	RRR	*95% Cis*	p-value	RRR	*95% Cis*	p-value	RRR	*95% Cis*	p-value
**Gender: Women (vs men)**	0.95	(*0.83;1.1*)	0.516	**1**.**22**	(***1.12;1.33*)**	**<0**.**001**	**1**.**47**	(***1.25;1.73*)**	**<0**.**001**
**Age: 18–29 years (vs 60+ years)**	**3**.**45**	(***2.71;4.39*)**	**<0**.**001**	**2**.**19**	(***1.88;2.54*)**	**<0**.**001**	**4**.**31**	(***3.34;5.57*)**	**<0**.**001**
**Age: 30 to 45 years (vs 60+ years)**	**2**.**35**	(***1.89;2.93*)**	**<0**.**001**	**1**.**80**	(***1.58;2.06*)**	**<0**.**001**	**2**.**68**	(***2.11;3.41*)**	**<0**.**001**
**Age: 46 to 59 years (vs 60+ years)**	**1**.**93**	(***1.6;2.32*)**	**<0**.**001**	**1**.**52**	(***1.36;1.69*)**	**<0**.**001**	**1**.**76**	(***1.43;2.17*)**	**<0**.**001**
**Ethnicity: Black, Asian, Minority (vs White)**	1.02	(*0.81;1.28*)	0.893	**1**.**29**	(***1.13;1.47*)**	**<0**.**001**	0.97	(*0.75;1.24*)	0.782
**Education: Low (vs High)**	1.16	(*0.97;1.39*)	0.111	1.08	(*0.97;1.21*)	0.148	**1**.**44**	(***1.19;1.75*)**	**<0**.**001**
**Education: Medium (vs High)**	**1**.**24**	(***1.06;1.46*)**	**0**.**007**	0.93	(*0.84;1.02*)	0.140	1.05	(*0.88;1.25*)	0.581
**Income: <£30,000 (vs >£30,000)**	**1**.**82**	(***1.56;2.12*)**	**<0**.**001**	**1**.**39**	(***1.26;1.52*)**	**<0**.**001**	**1**.**39**	(***1.18;1.62*)**	**<0**.**001**
**Income: prefer not to say (vs >£30,000)**	**1**.**43**	(***1.15;1.78*)**	**0**.**001**	1.08	(*0.94;1.25*)	0.251	**0**.**67**	(***0.5;0.89*)**	**0**.**005**
**Alone (vs With others, no children)**	**1**.**57**	(***1.33;1.85*)**	**<0**.**001**	**1**.**36**	(***1.23;1.51*)**	**<0**.**001**	**1**.**38**	(***1.15;1.67*)**	**0**.**001**
**Living with others, with children (vs Others, no children)**	1.04	(*0.87;1.23*)	0.672	1.06	(*0.95;1.17*)	0.309	**1**.**29**	(***1.08;1.54*)**	**0**.**005**
**Mental health diagnosis (vs none)**	**6**.**62**	(***5.77;7.61*)**	**<0**.**001**	**3**.**05**	(***2.77;3.36*)**	**<0**.**001**	**2**.**84**	(***2.42;3.34*)**	**<0**.**001**
**Carer (vs not a carer)**	**1**.**41**	(***1.19;1.67*)**	**<0**.**001**	**1**.**26**	(***1.13;1.4*)**	**<0**.**001**	1.11	(*0.92;1.35*)	0.279
**Keyworker (vs not a keyworker)**	1.04	(*0.89;1.23*)	0.609	0.96	(*0.87;1.06*)	0.467	1.08	(*0.92;1.28*)	0.357
**Long-term health condition (vs none)**	**1**.**98**	(***1.73;2.26*)**	**<0**.**001**	**1**.**56**	(***1.43;1.69*)**	**<0**.**001**	**1**.**42**	(***1.23;1.65*)**	**<0**.**001**
**Overcrowded living (vs not)**	**1**.**25**	(***1.04;1.5*)**	**0**.**019**	1.07	(*0.94;1.21*)	0.291	1.08	(*0.88;1.32*)	0.476
**Urban (vs Rural)**	1.08	(*0.95;1.23*)	0.249	**1**.**09**	(***1;1.18*)**	**0**.**048**	0.95	(*0.82;1.09*)	0.436
**Social: every day (vs once/twice a week)**	0.94	(*0.74;1.21*)	0.646	1.08	(*0.94;1.25*)	0.269	**1**.**36**	(***1.09;1.69*)**	**0**.**007**
**Social: three/four times a week (vs once/twice a week)**	0.88	(*0.73;1.06*)	0.170	0.97	(*0.87;1.08*)	0.531	0.85	(*0.71;1.03*)	0.097
**Social: once/twice a month (vs once/twice a week)**	0.98	(*0.82;1.17*)	0.843	1.05	(*0.94;1.18*)	0.348	0.93	(*0.77;1.13*)	0.479
**Social: less once month (vs once/twice a week)**	**1**.**71**	(***1.43;2.06*)**	**<0**.**001**	**1**.**31**	(***1.15;1.48*)**	**<0**.**001**	0.92	(*0.72;1.16*)	0.476
**Personality: Neuroticism**	**1**.**23**	(***1.21;1.25*)**	**<0**.**001**	**1**.**15**	(***1.14;1.16*)**	**<0**.**001**	**1**.**13**	(***1.11;1.15*)**	**<0**.**001**
**Personality: Extraversion**	**0**.**98**	(***0.96;0.99*)**	**0**.**003**	0.99	(*0.98;1*)	0.134	1.01	(*0.99;1.02*)	0.548
**Personality: Openness**	**1**.**05**	(***1.03;1.07*)**	**<0**.**001**	**1**.**05**	(***1.03;1.06*)**	**<0**.**001**	**1**.**07**	(***1.04;1.09*)**	**<0**.**001**
**Personality: Agreeableness**	1.00	(*0.98;1.02*)	0.967	1.01	(*0.99;1.02*)	0.292	1.00	(*0.98;1.02*)	0.896
**Personality: Conscientiousness**	**0**.**94**	(***0.92;0.96*)**	**<0**.**001**	**0**.**95**	(***0.94;0.96*)**	**<0**.**001**	**0**.**95**	(***0.93;0.98*)**	**<0**.**001**

Notes: RRR = relative risk ratio; 95%CIs = 95% confidence intervals. Figures in bold are statistically significant at p < 0.05.

#### Anxiety trajectories

Findings indicate that class 1 were typically older than the other classes, and that classes 3 (moderate symptoms), 4 (relief after lockdown) and 5 (severe initial anxiety which decreased) were more likely to be female than members of class 1. Class 3 were also more likely to be from BAME communities, and live in overcrowded accommodation, while class 4 were more likely to have low levels of education attainment, live with children or live alone, and to be considered a keyworker, compared to class 1. All classes were more likely to report both physical and mental health conditions compared to class 1, and mental health diagnoses were very prevalent in class 2 which might explain their higher symptom scores through the study period. Class 1 were the least likely to report being in the low-income group, and were less likely to be living alone than classes 3 and 4. Differences in personality characteristics were also observed, as higher scores on the neuroticism and openness subscales of the BFI were associated with being members of all classes other than class 1, with neuroticism scores particularly higher for classes 2 and 5. Extraversion was higher for classes 3, 4 and 5, while agreeableness was lower for members of class 2 and conscientiousness was higher for classes 2 and 5, but lower for class 3 compared to class 1.

Further analyses comparing characteristics of classes with similar intercepts (classes 2 and 5, as well as classes 3 and 4) were conducted using logistic regression (online Supplementary eTable 4). Class 2 individuals were less likely to be female, keyworkers, have had daily social contact before lockdown, and have lower extraversion scores, as well as being more likely to have a previous mental health diagnosis compared to class 5. Class 4 (relief after lockdown) participants were more likely to be female, younger, have lower educational attainment, be a keyworker, and have higher neuroticism, extraversion, and conscientiousness scores compared to class 3.

#### Depression trajectories

As found with the anxiety trajectories, depression class 1 were older than the other classes, were less likely to be female compared to class 3 (moderate severity) and class 4 (relief after lockdown), and class 3 consisted of more BAME group members. Class 1 were also less likely to report physical health or previous mental health diagnoses, to report a low income, or to be living alone compared to the other classes. Those in classes 2 and 3 were more likely to report being a carer, while overcrowding was more likely for class 2. Class 4 were more likely to report daily contact with others before the pandemic, whereas classes 2 and 3 were more likely to report socialising less than once a month. Neuroticism and openness subscales were lower for class 1 and conscientious was higher, whereas extraversion was found to be lower in class 2.

Further analyses comparing characteristics of classes 3 and 4 participants found that class 4 individuals were more likely to be younger and have lower educational attainment, and class 3 individuals were more likely to have social contact less than once a month (see online Supplementary eTable 4).

## Discussion

Findings from this study indicate that most participants reported low-level symptoms of depression and anxiety during the first 16 weeks of national lockdown in England, with further improvements over time. However, a considerable number (27–28%) reported scores suggestive of likely clinical disorder in the initial weeks of lockdown, and for some people these symptoms did not reduce over time. The easing of national restrictions was associated with observable decreases in symptoms for a small group of participants (around 4%) in both the anxiety and depression symptom analyses, although the speed at which symptoms decreased appeared slow after the easing of lockdown. Additionally, one group of participants experienced observable increases in symptoms even after the easing of lockdown. A number of participant characteristics were associated with being in these different trajectories, and notably being younger, having a previous mental health diagnosis, or a physical health condition, were associated with a lower likelihood of being in the low symptom severity subgroup.

The results of this study support previous analyses showing that on average there were improvements in depression and anxiety symptomatology across lockdown and the following weeks (Fancourt et al., 2020a). However, the modelling approach employed in the current study identified distinct patterns of change in symptom severity, highlighting that there are sub-groups of the population that did not follow this declining trend in symptoms. Instead we identified sub-groups with either moderate or severe levels of symptoms throughout, worsening of symptoms during lockdown but improvements as lockdown eased and (for anxiety), severe initial symptoms that improved quickly during lockdown.

When considering the risk factors associated with membership of any class other than the largest, lowest symptom class (class 1), a number of the participant factors identified in this analysis echoed those reported in recent research conducted during the pandemic. Being younger and female has been associated with increased depression and anxiety in a number of studies (Fancourt et al., 2020a; Shevlin et al., 2020; Xiong et al., 2020), while lower income, having children and physical health concerns have also been implicated in increased risk (Every-Palmer et al., 2020; McGinty, Presskreischer, Anderson, Han, & Barry, 2020; Shevlin et al., 2020). Many of these also reiterate risk factors for poorer mental health outside of pandemic circumstances (Allen, Balfour, Bell, & Marmot, 2014; Barnett et al., 2012; Riecher-Rössler, 2017).

In contrast to other studies, some socio-demographic groups were at particular risk of adverse mental health experiences here. Those in class 2 reported severe symptoms throughout the study period that continued to increase. They were more likely to have had a diagnosed mental disorder in the past and may require additional support for their mental health. The trajectory for this group may reflect the reduced access, availability or uptake of care from mental health services during the pandemic, and indeed there have been considerably fewer referrals to such services in England in this time (Saunders et al., 2021; Tromans et al., 2020). Worsening symptoms were also found for class 4 (although they started from a lower initial symptom score), but this group showed improvements once lockdown eased. Class 4 differed from class 2 as they were less likely to report being on a lower income or to have had a previously diagnosed mental or physical health condition. Class 4 were also more likely to have had degree-level education, and on average they had lower neuroticism and higher extraversion scores than class 2, which may explain why class 4 showed most benefit following the easing of lockdown.

Although the form of four trajectory classes of anxiety and depressive symptoms was similar, there were some differences in characteristics associated with these classes. For example, living alone and lower extraversion scores were associated with depression class 2, but not anxiety class 2, and more frequent social contact before the pandemic was associated with depression class 4 but not anxiety class 4. In addition, there was one trajectory class unique to anxiety symptoms, characterised by severe initial anxiety followed by substantial improvements during lockdown. Participants in this class were more likely to be younger, female, sociable and outgoing, which might represent people with significant concerns around the uncertainty of how lockdown would impact their daily lives, as well as concerns over the risks from the virus. Keyworkers were also more likely to be in this group, suggesting that initial concern during the pandemic may have been alleviated from the routine of more normal working patterns compared to individuals who were furloughed or lost their jobs. Anticipating this pattern in future pandemics or future periods of lockdown during the ongoing pandemic, especially among keyworkers, could enable the provision of additional support and resources to this group to try and reduce their psychological reactivity (Greenberg et al., 2020).

### Limitations

We have presented results from a very large stratified sample with high degrees of data-completion, and applied methods highlighting a number of trajectories of symptom change rather than a single average trajectory, providing more detailed information about how mental health has changed for different sub-groups. The sample included was not representative of the UK general population and therefore these trajectories may not generalise to the whole population. However, greater weight was given to those under-represented in this sample bringing estimates closer to those which may be expected in the wider population. We made data being completed at least six times during the study period an inclusion criterion. This resulted in the loss of nearly two-thirds of the COVID Social Study participants. This was necessary to perform the analyses presented here, but may have introduced a number of selection biases. In addition, we modelled anxiety and depression symptoms separately in analyses in order to detect differences, despite the high correlation between these scores in this study, and others. Although we have not compared the simultaneous change in depression and anxiety scores, the identification of an anxiety trajectory that was not identified in the depression analysis (GAD-7 class 5) suggests that modelling them together would lose this distinction. The analyses focused on changes in symptoms before, during and after the easing of national lockdown restrictions and a number of important covariates were considered, but residual confounding cannot be ruled out. Of note, severity scores before the pandemic were not available. Finally, although trajectories were identified during the initial 16 weeks of the pandemic, it is unknown whether these trajectories re-emerged, including for the same people, in subsequent lockdowns.

## Conclusions

Nearly 30 percentage of respondents experienced symptom trajectories that did not fit with the average or majority group, highlighting the importance of considering differential symptom trajectories. This study has allowed us to understand the nuance in these symptom trajectories. We have highlighted a number of patient characteristics associated with these non-normative curves. In particular, young, female, sociable and outgoing people, as well as keyworkers, appeared to experience severe levels of anxiety pre-lockdown which rapidly decreased during the period of restrictions. Furthermore, younger individuals with lower incomes and a previous mental health diagnosis were more likely to have higher initial levels of symptoms that increased steadily during and after the easing of restrictions. Recognising the likely symptom trajectories for such groups may allow for targeted care or interventions, ensuring that those in the former group are experiencing the predicted reductions in symptoms, and if not, then offering treatment to address any mental health problems. For the latter group, ensuring that they are aware of options to access support should they experience the predicted increases in symptoms, while periods of lockdown or other restrictions continue to be applied and relaxed throughout this pandemic or those to come in future, may be important to mitigate their predicted deleterious prognosis.

## Supplementary material

For supplementary material accompanying this paper visit http://dx.doi.org/10.1017/S0033291721000957. click here to view supplementary material
