# Supplementary material for: Understanding different trajectories of mental health across the general population during the COVID-19 pandemic
Source: Psychol Med. 2021 Mar 3:1–9. doi: 10.1017/S0033291721000957 (PMC8007951; doi:10.1017/S0033291721000957)
Supplement: Supplementary file 1 [file S0033291721000957sup001.docx]

Supplementary materials to:

Understanding different trajectories of mental health across the general population during the COVID-19 pandemic.

**Content:**

Appendix A: Study description and participant flow (Page 2)

Appendix B: Measures and participant characteristics (Page 4)

Appendix C: Model selection information (Page 6)

Appendix D: Descriptive statistics of trajectories. (Page 8)

Appendix E: Logistic regression analyses. (Page 11)

References (Page 13).

**Appendix A: Study description and participant flow.**

The COVID-19 Social Study commenced on March 21^st^ 2020 and involves online weekly data collection from participants for the duration of the COVID-19 pandemic in the UK. The study is not random and therefore is not representative of the UK population, but it does contain a well-stratified sample that was recruited using three primary approaches. First, convenience sampling was used, including promoting the study through existing networks and mailing lists (including large databases of adults who had previously consented to be involved in health research across the UK), print and digital media coverage, and social media. Second, more targeted recruitment was undertaken focusing on (i) individuals from a low-income background, (ii) individuals with no or few educational qualifications, and (iii) individuals who were unemployed. Third, the study was promoted via partnerships with third sector organisations to vulnerable groups, including adults with pre-existing mental health conditions, older adults, carers, and people experiencing domestic violence or abuse. The study was approved by the UCL Research Ethics Committee [12467/005] and all participants gave informed consent.

Following the announcement of the national UK lockdown on March 23^rd^ 2020, the guidance was eased on May 10^th^ 2020 in the England with the change of the “stay at home” slogan to “stay alert” which was accompanied by requests for those who could not work at home to travel to workplaces and encouraging unlimited exercise outside. To model changes in trajectories before and after this May 10^th^ date we further selected cases who provided mental health data for at least three time points between March 23^rd^ and May 10^th^, as well as providing data for three or more times in the first eight weeks after the easing of restrictions. From a total of 57,999 participants whose data were available within these dates, 23,802 did not provide data for three time points in the first eight weeks and a further 11,626 did not provide data for three time points in the second eight-week period and were therefore excluded. Of the remaining 22,571, 633 did not have complete data on gender, age, ethnicity, local level deprivation and level of educational attainment and therefore could not be weighted in analyses, resulting in a study sample size of 21,938 participants (flow diagram presented in eFigure1 below).


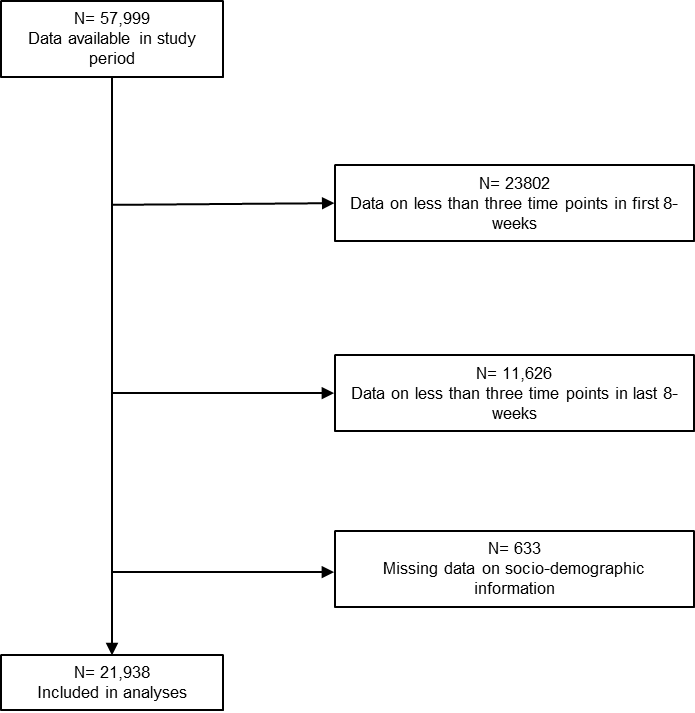


***eFigure1. Participant flow diagram for this study***

**Appendix B: Measures and participant characteristics**

# Measures

## Depression

Depressive symptoms were measured using the Patient Health Questionnaire nine-items (PHQ-9) (Kroenke, Spitzer, & Williams, 2001); a standard instrument for screening for depression in primary care as well as research. All nine items of the questionnaire are scored 0-3 with response options ranging from “not at all” (0) to “nearly every day” (3). Scores of 0-4 are indicative of subclinical depression, 5-9 suggesting mild depression, 10-14 indicating moderate depression, 15-19 suggesting moderately severe depression, and scores of 20-27 suggesting severe depression (Kroenke et al., 2001).

## Anxiety

Symptoms of generalized anxiety were measured using the Generalized Anxiety Disorder scale seven-items (GAD-7) (Spitzer, Kroenke, Williams, & Löwe, 2006); a well-validated tool used to screen for generalized anxiety disorder in clinical practice and research. The seven items of the scale are scored using the same response options as those for the PHQ-9. Scores can be banded as follows: 0-4 is considered subclinical, 5-9 is considered mild, between 10-14 is moderate and above 15 is considered severe (Spitzer et al., 2006). However, in clinical settings such as routine psychological treatment services a score of 8 or above is often considered to be above the clinical threshold for generalized anxiety (NHS, 2018; Saunders et al., 2020).

Details on participant sociodemographic and personality factors are presented in eTable1 below.

**eTable1. Description and categorisation of participant characteristics.**

| **Variable** | **Description** | **Categories used in analysis** |
| --- | --- | --- |
| Gender | Gender of participant | Grouped in "Women" or "Men" |
| Age | Self-reported age | Four categories: 18-29 years; 30-45 years; 46-59 years; 60+ years old |
| Ethnicity | Self-reported ethnicity | "White" or "Black, Asian and minority ethnic (BAME) groups" |
| Income | Household income | "Low income" defined as <£30,000 per year or "High income" defined as >=£30,000 per year. Responses "prefer not to say" were treated as further category. |
| Education | Education level attained | "General Certificate of Secondary Education (GCSE) or below" (low); "A-levels or equivalent" (medium); "Undergraduate degree or above" (high) |
| Living situation | Living arrangements with others | "Alone"; "With others including children"; "With others not including any children" |
| Local area | Population density of local area | "Urban"; "Rural" |
| Over-crowed | Whether less than 1 room per person in household | "Yes" or "no" |
| Keyworker | Whether participant's job is classed as key or critical worker (e.g. health and social care worker). | "Yes" or "no" |
| Carer | Whether participant has caring responsibilities | "Yes" or "no" |
| Mental health condition | Whether participant has previously been diagnosed with a mental health condition | "Yes" or "no" |
| Physical health condition | Whether participant has previously been diagnosed with a chronic physical health condition | "Yes" or "no" |
| Previous social contact | Response to "Usually in your life, how often to meet up with people face to face socially" | Five response categories: "Every day"; "Three or more times a week"; "Once or twice a week"; "Once or twice a month" ;"Less than once a month" |
| Big Five Personality | Subscale scores on the BFI-2 | Five subscale scores: "Neuroticism"; "Extraversion"; "Openness"; "Agreeableness"; "Conscientiousness" |

**Appendix C: Model selection information**

In order to identify the best fitting growth mixture model solution, model fit was compared using the Vuong-Lo-Medell-Rubin Likelihood Ratio Test (VLMR-LRT) (Lo, Mendell, & Rubin, 2001), the Akaike Information Criterion (AIC), Bayesian Information Criterion (BIC), and entropy values. The VLMR-LRT is a comparison between one model with K-classes, and the K-1 model, with a p-value < 0.05 indicating that the K model fits the data better than the K-1 model. Lower AIC and BIC values for one model compared to another indicate better model fit, whereas higher entropy values indicate higher accuracy in classification for the model (Geiser, 2013; Saunders et al., 2019). There was no prior hypothesis on the expected number of classes so the GMM was first conducted with a two-class model (identifying two classes) and then increasing the number of classes by one each time until the VLMR-LRT became non-significant or any of the AIC or BIC values increased compared to the previous class solution, as is standard for GMM methods (Musliner et al., 2016; Nylund, Asparouhov, & Muthén, 2007; Saunders et al., 2019).

Model fit statistics are presented in eTable2 below. Results of the GMMs showed that the GAD-7 class solutions showed decreasing AIC and BIC values, but as the VLMR-LRT p-value was not significant (p=0.142) for the 6-class solution, the 5-class solution was chosen. Entropy values were similar across classes. For the PHQ-9 models, the VLMR-LRT was significant until the 5-class solution (p=0.23), which when considered alongside the AIC, BIC and entropy values resulted in the 4-class solution being accepted to describe classes of depression symptom change.

**eTable2. Model fit statistics for GAD-7 and PHQ-9 GMMs.**

| **Anxiety symptoms (GAD-7)** | | | | | | | |
| --- | --- | --- | --- | --- | --- | --- | --- |
| **Class Solution** | **Log-Likelihood** | **AIC** | **BIC** | **Adj-BIC** | **VLMR-LRT p-value** | **Entropy** | **% individuals per class** |
| 2-Class | -655074 | 1310205 | 1310437 | 1310345 | <0.001 | 0.931 | 13/87 |
| 3-Class | -652859 | 135784 | 1306048 | 1305943 | <0.001 | 0.91 | 77/7/17 |
| 4-Class | -651361 | 1302797 | 1303093 | 1302975 | 0.005 | 0.909 | 6/73/16/4 |
| **5-Class** | **-650226** | **1300534** | **1300862** | **1300731** | **0.028** | **0.909** | **14/4/6/3/72** |
| 6-Class | -649269 | 1298628 | 1298988 | 1298845 | 0.142 | 0.906 | 3/16/8/3/66/5 |
| **Depression symptoms (PHQ-9)** | | | | | | | |
| **Class Solution** | **Log-Likelihood** | **AIC** | **BIC** | **Adj-BIC** | **VLMR-LRT p-value** | **Entropy** | **% individuals per class** |
| 2-Class | -690512 | 1381081 | 1381313 | 1381221 | <0.001 | 0.902 | 14/86 |
| 3-Class | -689082 | 1378229 | 1378493 | 1378388 | 0.002 | 0.875 | 74/19/6 |
| **4-Class** | **-687894** | **1375862** | **1376158** | **1376040** | **0.007** | **0.88** | **4/73/6/17** |
| 5-Class | -687169 | 1374420 | 1374748 | 1374618 | 0.230 | 0.884 | 72/6/5/16/2 |
| 6-Class | -686423 | 1372935 | 1373295 | 1373152 | 0.297 | 0.877 | 14/5/2/3/5/70 |

**Appendix D: Descriptive statistics of trajectories.**

**eTable3. Descriptive statistics of the identified GAD-7 and PHQ-9 classes.**

|  | **GAD-7 Classes** | | | | | **PHQ-9 Classes** | | | |
| --- | --- | --- | --- | --- | --- | --- | --- | --- | --- |
|  | **Class 1** | **Class 2** | **Class 3** | **Class 4** | **Class 5** | **Class 1** | **Class 2** | **Class 3** | **Class 4** |
|  | **N (%)** | **N (%)** | **N (%)** | **N (%)** | **N (%)** | **N (%)** | **N (%)** | **N (%)** | **N (%)** |
| **Gender** | | | | | | | | | |
| Women | 8389(53) | 990(71) | 2022(67) | 685(77) | 604(82) | 8642(54) | 868(65) | 2487(67) | 693(73) |
| Men | 7493(47) | 412(29) | 1000(33) | 210(23) | 134(18) | 7328(46) | 462(35) | 1206(33) | 253(27) |
| **Age in years** | | | | | | | | | |
| 18-29 | 1438(9) | 278(20) | 576(19) | 217(24) | 173(23) | 1546(10) | 273(21) | 638(17) | 224(24) |
| 30-45 | 2691(17) | 362(26) | 715(24) | 257(29) | 177(24) | 2796(18) | 316(24) | 844(23) | 247(26) |
| 46-59 | 5402(34) | 510(36) | 1060(35) | 290(32) | 260(35) | 5385(34) | 509(38) | 1328(36) | 300(32) |
| 60+ | 6350(40) | 251(18) | 671(22) | 132(15) | 127(17) | 6242(39) | 232(17) | 883(24) | 174(18) |
| **Ethnicity** | | | | | | | | | |
| White | 14606(92) | 1273(91) | 2673(88) | 794(89) | 691(94) | 14670(92) | 1219(92) | 3285(89) | 862(91) |
| BAME | 1276(8) | 129(9) | 349(12) | 102(11) | 47(6) | 1300(8) | 111(8) | 408(11) | 84(9) |
| **Household income** | | | | | | | | | |
| <£30,000 | 6169(39) | 771(55) | 1322(44) | 395(44) | 301(41) | 6002(38) | 760(57) | 1751(47) | 446(47) |
| >=£30,000 | 8098(51) | 474(34) | 1385(46) | 431(48) | 367(50) | 8329(52) | 415(31) | 1571(43) | 439(46) |
| Prefer not to say | 1615(10) | 156(11) | 314(10) | 69(8) | 70(9) | 1639(10) | 154(12) | 370(10) | 61(6) |
| **Keyworker** | | | | | | | | | |
| No | 12799(81) | 1138(81) | 2395(79) | 663(74) | 536(73) | 12765(80) | 1072(81) | 2963(80) | 732(77) |
| Yes | 3082(19) | 263(19) | 627(21) | 233(26) | 201(27) | 3205(20) | 257(19) | 730(20) | 214(23) |
| **Education** | | | | | | | | | |
| GCSE or below | 4572(29) | 440(31) | 778(26) | 252(28) | 200(27) | 4521(28) | 386(29) | 1060(29) | 274(29) |
| A-levels or equivalent | 4886(31) | 489(35) | 911(30) | 242(27) | 255(35) | 4902(31) | 501(38) | 1098(30) | 284(30) |
| Degree or above | 6423(40) | 472(34) | 1333(44) | 402(45) | 283(38) | 6547(41) | 443(33) | 1535(42) | 388(41) |
| **Carer** | | | | | | | | | |
| No | 13637(86) | 1165(83) | 2524(84) | 765(85) | 624(85) | 13724(86) | 1102(83) | 3084(84) | 806(85) |
| Yes | 2245(14) | 236(17) | 498(16) | 131(15) | 114(15) | 2246(14) | 228(17) | 609(16) | 140(15) |
| **Living status** | | | | | | | | | |
| Alone | 3084(19) | 306(22) | 631(21) | 173(19) | 108(15) | 2909(18) | 352(26) | 846(23) | 194(21) |
| With others, no children | 9671(61) | 772(55) | 1703(56) | 483(54) | 441(60) | 9820(61) | 703(53) | 2048(55) | 499(53) |
| With others, with children | 3126(20) | 324(23) | 689(23) | 240(27) | 188(26) | 3241(20) | 275(21) | 798(22) | 253(27) |
| **Overcrowded** | | | | | | | | | |
| No | 14437(91) | 1173(84) | 2555(85) | 766(86) | 639(87) | 14442(90) | 1119(84) | 3203(87) | 806(85) |
| Yes | 1445(9) | 229(16) | 467(15) | 129(14) | 99(13) | 1528(10) | 211(16) | 490(13) | 139(15) |
| **Urban/Rural** | | | | | | | | | |
| Rural | 7765(49) | 595(42) | 1320(44) | 387(43) | 316(43) | 7792(49) | 568(43) | 1595(43) | 429(45) |
| Urban | 8116(51) | 806(58) | 1703(56) | 508(57) | 422(57) | 8178(51) | 762(57) | 2098(57) | 517(55) |
| **Diagnosed mental illness** | | | | | | | | | |
| No | 14558(92) | 548(39) | 1994(66) | 560(63) | 510(69) | 14645(92) | 502(38) | 2393(65) | 629(67) |
| Yes | 1324(8) | 854(61) | 1028(34) | 335(37) | 227(31) | 1324(8) | 827(62) | 1300(35) | 316(33) |
| **Long-term physical health condition** | | | | | | | | | |
| No | 9415(59) | 608(43) | 1660(55) | 525(59) | 423(57) | 9650(60) | 559(42) | 1884(51) | 538(57) |
| Yes | 6466(41) | 793(57) | 1362(45) | 371(41) | 315(43) | 6319(40) | 771(58) | 1809(49) | 407(43) |
| **Previous social contact frequency** | | | | | | | | | |
| Every day | 1646(10) | 114(8) | 288(10) | 108(12) | 106(14) | 1668(10) | 101(8) | 361(10) | 131(14) |
| Three or more times a week | 3906(25) | 269(19) | 611(20) | 184(21) | 171(23) | 3946(25) | 228(17) | 775(21) | 191(20) |
| Once or twice a week | 5525(35) | 423(30) | 1056(35) | 331(37) | 217(29) | 5552(35) | 426(32) | 1232(33) | 342(36) |
| Once or twice a month | 3010(19) | 290(21) | 612(20) | 147(16) | 160(22) | 3033(19) | 257(19) | 749(20) | 181(19) |
| Less than once a month | 1795(11) | 305(22) | 454(15) | 126(14) | 84(11) | 1770(11) | 318(24) | 575(16) | 101(11) |
| **Big Five personality factor** | **M (SD)** | **M (SD)** | **M (SD)** | **M (SD)** | **M (SD)** | **M (SD)** | **M (SD)** | **M (SD)** | **M (SD)** |
| Neuroticism | 10(3.9) | 16(3.8) | 13.7(3.7) | 14.1(3.7) | 15(3.6) | 10.2(4) | 15.2(4.1) | 13.4(4) | 13.3(4.1) |
| Extraversion | 12.8(4.2) | 11(4.5) | 12.2(4.3) | 12.8(4.4) | 12.8(4.5) | 12.9(4.2) | 10.9(4.4) | 12(4.4) | 12.6(4.4) |
| Openness | 14.8(3.2) | 15(3.8) | 15.1(3.4) | 15.2(3.5) | 15.3(3.5) | 14.8(3.2) | 14.9(3.8) | 15.1(3.3) | 15.3(3.3) |
| Agreeableness | 15.5(3) | 15.2(3.7) | 15.5(3.1) | 15.5(3.1) | 15.7(3.2) | 15.5(3) | 15.2(3.7) | 15.4(3.1) | 15.5(3.2) |
| Conscientiousness | 15.9(2.9) | 15.5(3.5) | 15.4(3.1) | 15.8(3.2) | 16.1(3.2) | 16(2.8) | 14.8(3.5) | 15.3(3.1) | 15.5(3.1) |

**Appendix E: Logistic regression analyses.**

**eTable4. Predictors of membership to Class 2 vs Class 5 (GAD-7) and Class 3 vs Class 4 (GAD-7 & PHQ-9).**

|  | **GAD: Class 2 (vs Class 5)** | | |  | **GAD: Class 4 (vs Class 3)** | | |  | **PHQ: Class 4 (vs Class 3)** | | |
| --- | --- | --- | --- | --- | --- | --- | --- | --- | --- | --- | --- |
|  | **OR** | ***95% CIs*** | **p-value** |  | **OR** | ***95% CIs*** | **p-value** |  | **OR** | ***95% CIs*** | **p-value** |
| **Gender: Women (vs men)** | **0.56** | ***(0.39;0.81)*** | **0.002** |  | **1.40** | ***(1.07;1.82)*** | **0.013** |  | 1.21 | *(0.94;1.54)* | 0.136 |
| **Age: 18-29 years (vs 60+ years)** | 0.92 | *(0.56;1.51)* | 0.750 |  | **2.02** | ***(1.37;2.99)*** | **<0.001** |  | **2.01** | ***(1.39;2.89)*** | **<0.001** |
| **Age: 30 to 45 years (vs 60+ years)** | 1.08 | *(0.7;1.67)* | 0.731 |  | **1.81** | ***(1.29;2.52)*** | **0.001** |  | **1.50** | ***(1.09;2.08)*** | **0.014** |
| **Age: 46 to 59 years (vs 60+ years)** | 0.93 | *(0.64;1.36)* | 0.708 |  | 1.34 | *(0.98;1.83)* | 0.071 |  | 1.15 | *(0.86;1.54)* | 0.347 |
| **Ethnicity: Black, Asian, Minority (vs White)** | 1.57 | *(0.89;2.78)* | 0.117 |  | 0.92 | *(0.63;1.34)* | 0.670 |  | 0.75 | *(0.5;1.11)* | 0.149 |
| **Education: Low (vs High)** | 1.12 | *(0.78;1.59)* | 0.548 |  | **1.35** | ***(1.01;1.8)*** | **0.046** |  | **1.38** | ***(1.05;1.82)*** | **0.021** |
| **Education: Medium (vs High)** | 0.93 | *(0.68;1.28)* | 0.666 |  | 0.95 | *(0.73;1.23)* | 0.673 |  | 1.12 | *(0.88;1.43)* | 0.345 |
| **Income: <£30,000 (vs >£30,000)** | 1.36 | *(1;1.85)* | 0.053 |  | 1.09 | *(0.87;1.38)* | 0.458 |  | 0.99 | *(0.79;1.24)* | 0.901 |
| **Income: prefer not to say (vs >£30,000)** | 1.39 | *(0.9;2.14)* | 0.137 |  | 0.76 | *(0.52;1.13)* | 0.174 |  | **0.63** | ***(0.41;0.95)*** | **0.027** |
| **Alone (vs With others, no children)** | 1.29 | *(0.89;1.87)* | 0.185 |  | 1.05 | *(0.79;1.4)* | 0.721 |  | 1.04 | *(0.79;1.36)* | 0.785 |
| **Living with others, with children (vs Others, no children)** | 1.06 | *(0.76;1.47)* | 0.733 |  | 1.25 | *(0.97;1.6)* | 0.083 |  | 1.23 | *(0.97;1.57)* | 0.094 |
| **Mental health diagnosis (vs none)** | **2.72** | ***(2.05;3.6)*** | **<0.001** |  | 1.12 | *(0.9;1.41)* | 0.318 |  | 0.93 | *(0.74;1.16)* | 0.494 |
| **Carer (vs not a carer)** | 1.31 | *(0.94;1.83)* | 0.105 |  | 0.83 | *(0.64;1.07)* | 0.147 |  | 0.88 | *(0.68;1.15)* | 0.341 |
| **Keyworker (vs not a keyworker)** | **0.70** | ***(0.51;0.96)*** | **0.025** |  | **1.31** | ***(1.03;1.66)*** | **0.025** |  | 1.14 | *(0.91;1.44)* | 0.253 |
| **Long-term health condition (vs none)** | 1.24 | *(0.95;1.62)* | 0.119 |  | 0.96 | *(0.77;1.2)* | 0.740 |  | 0.90 | *(0.73;1.11)* | 0.329 |
| **Overcrowded living (vs not)** | 1.41 | *(0.98;2.03)* | 0.062 |  | 0.77 | *(0.56;1.05)* | 0.100 |  | 1.01 | *(0.75;1.36)* | 0.956 |
| **Urban (vs Rural)** | 0.95 | *(0.73;1.23)* | 0.697 |  | 0.96 | *(0.77;1.19)* | 0.699 |  | 0.84 | *(0.69;1.03)* | 0.097 |
| **Social: every day (vs once/twice a week)** | **0.57** | ***(0.35;0.93)*** | **0.024** |  | 1.19 | *(0.83;1.7)* | 0.340 |  | 1.22 | *(0.88;1.71)* | 0.236 |
| **Social: three/four times a week (vs once/twice a week)** | 0.86 | *(0.6;1.23)* | 0.420 |  | 0.95 | *(0.72;1.25)* | 0.699 |  | 0.87 | *(0.67;1.13)* | 0.294 |
| **Social: once/twice a month (vs once/twice a week)** | 0.90 | *(0.64;1.29)* | 0.574 |  | 0.77 | *(0.58;1.03)* | 0.074 |  | 0.88 | *(0.67;1.15)* | 0.350 |
| **Social: less once month (vs once/twice a week)** | 1.24 | *(0.82;1.86)* | 0.307 |  | 1.01 | *(0.72;1.42)* | 0.959 |  | **0.68** | ***(0.48;0.97)*** | **0.032** |
| **Personality: Neuroticism** | 1.03 | *(0.99;1.07)* | 0.112 |  | **1.03** | ***(1;1.06)*** | **0.038** |  | 0.99 | *(0.96;1.01)* | 0.342 |
| **Personality: Extraversion** | **0.96** | ***(0.93;0.99)*** | **0.020** |  | **1.03** | ***(1.01;1.06)*** | **0.015** |  | 1.02 | *(0.99;1.04)* | 0.246 |
| **Personality: Openness** | 1.00 | *(0.96;1.04)* | 0.995 |  | 1.01 | *(0.98;1.05)* | 0.394 |  | 1.02 | *(0.99;1.05)* | 0.174 |
| **Personality: Agreeableness** | 0.97 | *(0.93;1.01)* | 0.193 |  | 0.98 | *(0.95;1.02)* | 0.279 |  | 0.99 | *(0.96;1.03)* | 0.662 |
| **Personality: Conscientiousness** | 1.01 | *(0.97;1.05)* | 0.659 |  | **1.04** | ***(1;1.08)*** | **0.032** |  | 1.00 | *(0.97;1.04)* | 0.827 |
| Notes: OR = Odds ratio; 95%CIs = 95% confidence intervals | |  |  |  |  |  |  |  |  |  |  |

**References**

Geiser, C. (2013). *Data analysis with Mplus*. New York: Guilford.

Kroenke, K., Spitzer, R. L., & Williams, J. B. W. (2001). The PHQ-9: Validity of a Brief Depression Severity Measure. *Journal of General Internal Medicine*, *16*, 606–613.

Lo, Y., Mendell, N. R., & Rubin, D. B. (2001). Testing the number of components in a normal mixture. *Biometrika*, *88*(3), 767–778.

Musliner, K. L., Munk-Olsen, T., Laursen, T. M., Eaton, W. W., Zandi, P. P., & Mortensen, P. B. (2016). Heterogeneity in 10-Year Course Trajectories of Moderate to Severe Major Depressive Disorder. *JAMA Psychiatry*, *73*(4), 346. https://doi.org/10.1001/jamapsychiatry.2015.3365

NHS. (2018). *Psychological Therapies, Annual Report on the use of IAPT Services*. *NHS Digital*. London.

Nylund, K. L., Asparouhov, T., & Muthén, B. O. (2007). Deciding on the number of classes in latent class analysis and growth mixture modeling: A Monte Carlo simulation study. *Structural Equation Modeling*, *14*(4), 535–569. https://doi.org/10.1080/10705510701575396

Saunders, R., Buckman, J. E. J., Cape, J., Fearon, P., Leibowitz, J., & Pilling, S. (2019). Trajectories of depression and anxiety symptom change during psychological therapy. *Journal of Affective Disorders*, *249*, 327–335. https://doi.org/10.1016/j.jad.2019.02.043

Saunders, R., Cape, J., Leibowitz, J., Aguirre, E., Jena, R., Cirkovic, M., … Buckman, J. E. J. (2020). Improvement in IAPT outcomes over time: Are they driven by changes in clinical practice? *Cognitive Behaviour Therapist*, *13*, e16. https://doi.org/10.1017/S1754470X20000173

Spitzer, R. L., Kroenke, K., Williams, J. B. W., & Löwe, B. (2006). A Brief Measure for Assessing Generalized Anxiety Disorder. *Archives of Internal Medicine*, *166*(10), 1092. https://doi.org/10.1001/archinte.166.10.1092
